# Immobilization of Cofactor Self-Sufficient Recombinant *Escherichia coli* for Enantioselective Biosynthesis of (*R*)-1-Phenyl-1,2-Ethanediol

**DOI:** 10.3389/fbioe.2020.00017

**Published:** 2020-02-21

**Authors:** Fei Peng, Hui-Hui Su, Xiao-Yang Ou, Zi-Fu Ni, Min-Hua Zong, Wen-Yong Lou

**Affiliations:** Laboratory of Applied Biocatalysis, School of Food Science and Engineering, South China University of Technology, Guangzhou, China

**Keywords:** (*R*)-1-phenyl-1, 2-ethanediol, 2-hydroxyacetophenone, (*2R*, *3R*)-butanediol dehydrogenase, asymmetric reduction, whole cell catalysis, immobilized cells

## Abstract

(*R*)-1-phenyl-1,2-ethanediol is an important synthon for the preparation of β-adrenergic blocking agents. This study identified a (*2R,3R*)-butanediol dehydrogenase (*Kg*BDH) from *Kurthia gibsonii* SC0312, which showed high enantioselectivity for production of (*R*)-1-phenyl-1,2-ethanediol by reduction of 2-hydroxyacetophenone. *Kg*BDH was expressed in a recombinant engineered strain, purified, and characterized. It showed good catalytic activity at pH 6–8 and better stability in alkaline (pH 7.5–8) than an acidic environment (pH 6.0–7.0), providing approximately 73 and 88% of residual activity after 96 h at pH 7.5 and 8.0, respectively. The maximum catalytic activity was obtained at 45°C; nevertheless, poor thermal stability was observed at >30°C. Additionally, the examined metal ions did not activate the catalytic activity of *Kg*BDH. A recombinant *Escherichia coli* strain coexpressing *Kg*BDH and glucose dehydrogenase (GHD) was constructed and immobilized via entrapment with a mixture of activated carbon and calcium alginate via entrapment. The immobilized cells had 1.8-fold higher catalytic activity than that of cells immobilized by calcium alginate alone. The maximum catalytic activity of the immobilized cells was achieved at pH 7.5, and favorable pH stability was observed at pH 6.0–9.0. Moreover, the immobilized cells showed favorable thermal stability at 25–30°C and better operational stability than free cells, retaining approximately 55% of the initial catalytic activity after four cycles. Finally, 81% yields (195 mM product) and >99% enantiomeric excess (*ee*) of (*R*)-1-phenyl-1,2-ethanediol were produced within 12 h through a fed-batch strategy with the immobilized cells (25 mg/ml wet cells) at 35°C and 180 rpm, with a productivity of approximately 54 g/L per day.

## Introduction

Chiral 1-phenyl-1,2-ethanediol (PED) plays an essential role in the synthesis of fine chemical compounds, pharmaceutical industry compounds, and liquid crystals (Cazetta et al., 2014; Li et al., 2014). The enantiomer (*R*)-PED is used as an important synthon for the fabrication of (*R*)-norfluoxetine, (*R*)-fluoxetine (Cui et al., 2017), and β-adrenergic blocking agents (Kamal et al., 2005). In recent decades, the preparation of chiral chemical compounds by biocatalysis has attracted considerable interest because of their high enantioselectivity, environmental friendliness, and mild reaction conditions (Ni and Xu, 2012; Choi et al., 2015; Lin and Tao, 2017). Compared with enzymatic catalysis, whole-cell biocatalysis has several advantages, including easy preparation, cofactor regeneration, and protection of intracellular enzymes (Wachtmeister and Rother, 2016). However, the biocatalytic reaction of wild strains was often confronted with some shortcomings owing to the low levels of functional enzymes, such as long reaction time, low levels of substrate, and high cell catalyst counts (Lü et al., 2007; Hu et al., 2010).

Some alcohol dehydrogenases from microorganisms have been reported to asymmetrically reduce 2-hydroxyacetophenone (HAP) to (*R*)-PED (Chen et al., 2015; Sudhakara and Chadha, 2017). *Kurthia gibsonii* SC0312 (*K. gibsonii* SC0312) isolated by our group is able to prepare chiral PED with high enantioselectivity (Peng et al., 2019). Nevertheless, the functional enzymes from *K. gibsonii* SC0312 for the preparation of chiral PED were yet unknown. Investigation of the functional enzymes and the enzymatic characteristics was therefore essential. *Escherichia coli* (*E. coli*) is a common host for the expression of heterologous genes because of its well-studied genetic background, rapid growth, and high expression levels (Lin and Tao, 2017; Wang P. et al., 2017). The use of alcohol dehydrogenases in oxidation–reduction reactions generally requires the participation of cofactors, such as NAD(P)^+^ or NAD(P)H (Kroutil et al., 2004). On the other hand, *E. coli* generally does not contain enough cofactors for oxidation–reduction reactions, and the use of additional cofactors decreases the cost efficiency. The construction of a cofactor regeneration system is capable of increasing cost efficiency by adding inexpensive cosubstrates (Liu et al., 2017). Glucose dehydrogenases (GHDs) are often used in the construction of enzyme-coupled systems (Hollmann et al., 2010) for the regeneration of either NADPH or NADH (Wichmann and Vasic-Racki, 2005).

Whole cells generally confront dissatisfactory usability and recyclability after operation in catalytic reaction (Wang L. et al., 2017). Cell immobilization technology may address the issues. Cell entrapment is a typical immobilization approach with advantages that include high cell density, easy recyclability, and facile preparation (dos Santos Belgrano et al., 2018). The use of calcium alginate for cell entrapment has attracted considerable attention because of its good biocompatibility and easy preparation (Ha et al., 2009). Most efforts have focused on enhancing the mechanical strength of calcium alginate beads, for instance, by forming a protective layer on the beads by using chitosan and dopamine (Chen et al., 2012; Kim et al., 2014) and introducing the supporting material polyvinyl alcohol (Wei et al., 2018). Another notable issue in cell entrapment is mass transfer limitation. However, few studies have focused on improving the catalytic rate of immobilized cells by entrapment.

**SCHEME 1:**
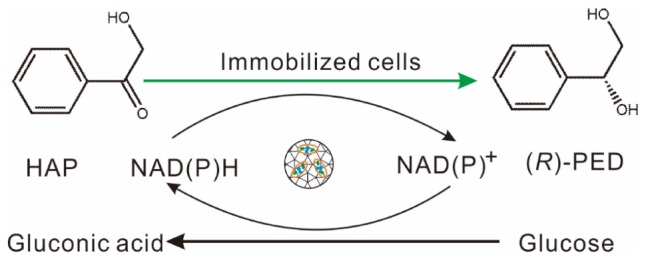
Biotransformation of HAP to (R)-PED by immobilized BL21(DE3)-pETduet1-*Kg*BDH-*Bs*GDH in buffer.

In this study, we discovered an alcohol dehydrogenase from *K. gibsonii* SC0312, which is used for the preparation of (*R*)-PED by the reduction of HAP with high enantioselectivity. The gene encoding the enzyme was first cloned and expressed in *E. coli* BL21(DE3) strain, and the enzymatic characteristics were assayed after purification by Ni-NTA agarose. Second, both the enzyme and the GHD were coexpressed in an *E. coli* BL21(DE3) engineered strain, and the recombinant cells were immobilized with a mixture of calcium alginate and active carbon. Finally, we constructed a highly enantioselective aqueous reaction system for the fabrication of (*R*)-PED by the reduction of HAP by using the immobilized cells coexpressing alcohol dehydrogenase and GDH (Scheme 1).

## Materials and Methods

### Biological and Chemical Materials

*Kurthia gibsonii* SC0312 and *Bacillus subtilis* 168 were stored in our laboratory. The host cells *E. coli* DH5α and *E. coli* BL21(DE3) as well as the plasmids pET28a and pETDuet1 were purchased from Novagen. The kits used for the recycling of genes and plasmids were obtained from Sangon Biotech (Shanghai, China). The restriction enzymes and the T4 DNA ligase were brought from Thermo Fisher Scientific^TM^. HAP (97%) was purchased from Aladdin (China). Other reagents were obtained from commercial sources. The clone and expression of genes as well as enzymatic purification are described in the Supplementary Material.

### Characteristics of the Functional Enzyme for the Asymmetric Reduction of HAP

The enzyme 2,3-butanediol dehydrogenase (*Kg*BDH) from *K. gibsonii* SC0312 was found to asymmetrically reduce HAP to (*R*)-PED. The HAP reduction activity of *Kg*BDH was assayed at different pH levels (pH 5–10) and temperatures (20–60°C) by determination of the absorbance of NADH at 340 nm. The reaction system encompassed PB (50 mM, pH 7.5), HAP 5 mM, NADH 0.2 mM, and an appropriate amount of *Kg*BDH. The reaction system without cofactors was set as the control group. The effects of metal ions (0.2 mM) on the *Kg*BDH activity were also evaluated, and the control group did not include the metal ions. The amount of *Kg*BDH oxidizing 1.0 μmol of cofactor per minute at 40°C and pH 7.5 was defined as one unit of enzyme activity. The protein level of the *Kg*BDH solution was measured with the Bradford method.

The pH stability and the thermal stability of *Kg*BDH were also assayed. The pH stability of the enzyme was measured by incubation in PB (pH 6.0–8.0, 4°C). The thermal stability of *Kg*BDH was measured by incubation in PB (50 mM, pH 7.5) within the temperature range of 20–50°C. The enzyme solution was regularly withdrawn for measurement of the reduction activity of *Kg*BDH during the incubation process. The kinetic parameters of *Kg*BDH for the reduction of HAP were measured at 40°C in PB (50 mM, pH 7.5). The levels of HAP ranged from 0.5 to 5 mM, and the concentration of NADH was at 0.2 mM.

### Cell Immobilization by Calcium Alginate and Activated Carbon

A recombinant *E. coli* BL21(DE3) strain with cofactor regeneration was constructed (shown in the Supplementary Material), which coexpressed *Kg*BDH and glucose dehydrogenase (*Bs*GDH) from *B. subtilis* 168. The *E. coli* BL21(DE3) cells coexpressing both *Kg*BDH and *Bs*GDH [*E. coli* BL21(DE3)-*Kg*BDH-*Bs*GDH] were first cultured overnight at 37°C and 160 rpm in LB broth containing 100 mg/L ampicillin. Then 1 ml of the overnight culture was inoculated into 100 ml fresh LB broth with 100 mg/L ampicillin, followed by culture at 37°C and 160 rpm. When the OD600 of the cell cultures reached 0.6–0.8, IPTG (0.1 mM) was injected to induce the expression of the target genes, followed by incubation at 20°C and 160 rpm for 20 h. The cells were collected by centrifugation (6,000 × *g*, 3 min) at 4°C and washed twice by normal saline. The cells were suspended in Tris–HCl buffer (50 mM, pH 7.5) until the immobilization of the cells. For cell immobilization, 1.0 g wet cells were mixed with 20 ml of sodium alginate (2%, w/v) containing active carbon granules (200 mg). Subsequently, the mixture was dripped into 0.2 mol/L CaCl_2_ with a needle to form beads. The formed beads were continuously hardened at 4°C for 3 h. Finally, the immobilized cells were stored at 4°C until use. The *E. coli* BL21(DE3)-*Kg*BDH-*Bs*GDH cells immobilized by calcium alginate (2%) were prepared as above. Comparisons were made among free cells, cells immobilized by calcium alginate, and cells immobilized by a mixture of activated carbon and calcium alginate. In addition, scanning electron microscopy (SEM) images of the cells immobilized by the mixture of activated carbon and calcium alginate were taken.

### Effects of pH and Temperature on the Immobilized Cells and Operational Stability

The catalytic activities of the immobilized cells for the reduction of HAP at different pH values and reaction temperatures were determined. Two buffers were applied to evaluate the effect of pH on the catalytic activity of the immobilized cells: sodium acetate (100 mM, pH 6.0–7.0) and Tris–HCl (50 mM, pH 7.5–9.0). The temperature ranged from 25 to 45°C. The reactions were conducted at 20 mM HAP, 40 mM glucose, 180 rpm, as well as the designed buffer pH and temperature. The catalytic rate was calculated according to the amount of PED generated after 30 min of reaction.

The pH stability and the thermal stability of the immobilized cells were also assayed. The immobilized cells were incubated in the reaction buffers above for 24 h at 4°C, and then the residual catalytic activity was analyzed. Similarly, the thermal stability of the immobilized cells preincubated in Tris–HCl (50 mM, pH 7.5) at the specified temperatures for 12 h was determined, and the catalytic activity was determined as described above.

The reusability of the immobilized cells was accessed by determining the residual activity for four reuse cycles. The adsorptive product on the surface of beads was wiped off using a Tris–HCl buffer (50 mM, pH 7.5). Meanwhile, to better minimize the influence of the residual product, a system consisted of Tris–HCl (50 mM, pH 7.5) and washed beads was employed to determine the residual amount of the product in the washed beads. Each cycle was conducted in a Tris–HCl buffer (50 mM, pH 7.5) under the designed conditions: 20 mM HAP, 40 mM glucose, 35°C, and 180 rpm for 3 h. The initial reaction rate was calculated according to the amount of (*R*)-PED after reaction for 30 min minus the residual amount of the product in the washed beads. Comparisons were made among free cells, cells immobilized by calcium alginate, and cells immobilized by a mixture of activated carbon and calcium alginate.

### Production of (*R*)-PED by Fed-Batch Feeding of HAP

The reaction system consisted of 4 ml Tris–HCl buffer (50 mM, pH 7.2), 80 mM HAP, 160 mM glucose, and immobilized cells (wet cell weight of 100 mg) and incubated at 35°C and 180 rpm; 320 mmol HAP and 640 mmol glucose were repeatedly added to the reaction system when HAP was nearly depleted. During the reaction process, sodium bicarbonate was used to neutralize the produced acids and to maintain the reaction system at a suitable pH level (pH 6.5–7.5). The levels of (*R*)-PED were monitored before supplementation with HAP during the reaction. The yield of the product was defined as the ratio of the amount of generated (*R*)-PED to that of the theoretical product. The enantiomeric excess (*ee*) of (*R*)-PED was calculated with the following equation:

$$e\,e=\frac{C_{R}-C_{S}}{C_{R}+C_{S}}\times 100\%$$

where *C*_R_ and *C*_S_ are the levels of (*R*)-PED and (*S*)-PED, respectively.

### Analytical Methods

The product (*R*)-PED concentration and the optical purity were analyzed by high-performance liquid chromatography (HPLC). The levels of (*R*)-PED were determined by Waters HPLC 1525 instrument equipped with a detector and XBridge-C8 column (4.6 mm × 250 mm, 5 μm, Waters, United States) using water and acetonitrile (6:4, v/v) as the mobile phase at 215 nm and 0.5 ml/min (Supplementary Figure S1a). The enantiomeric excess was monitored by Agilent HPLC 1100 instrument equipped with a UV detector at 215 nm, with an OB-H as the analytic column (4.6 mm × 150 mm, 5 μm, Agilent, United States) and hexane and isopropanol (9:1, v/v) as the mobile phase at 215 nm and 0.7 ml/min (Supplementary Figure S1b). The results are expressed as mean ± standard deviation, and all experiments were performed at least in duplicate.

## Results and Discussion

### Clone and Expression of Functional Genes From *K. gibsonii* SC0312

**FIGURE 1:**
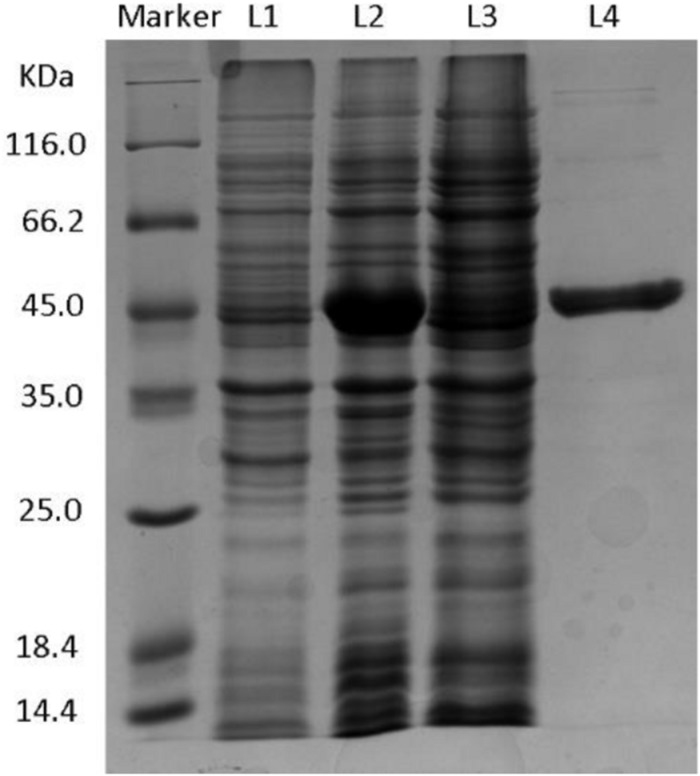
SDS-PAGE of *Kg*BDH. L1: Control, total proteins from BL21(DE3)-pET28a-*Kg*BDH strain without IPTG. L2: Whole cell, total proteins from BL21(DE3)-pET28a-*Kg*BDH strain with 0.1 mM IPTG. L3: Supernatant, soluble proteins from BL21(DE3)-pET28a-*Kg*BDH strain with 0.1 mM IPTG. L4: Purified *Kg*BDH.

SDS-PAGE was used for examining the expression of enzyme protein in the engineered strain. Figure 1 and Supplementary Figure S2 show that the eight putative enzymes were successfully expressed in the *E. coli* BL21(DE3) strain. On the basis of the analysis of the catalytic activity of whole cells and the crude enzyme solutions, only *Kg*BDH was able to reduce HAP, with high enantioselectivity of >99% for (*R*)-PED production (data not shown). The amino acid sequence analysis of *Kg*BDH is shown in Supplementary Figure S3. InterProScan prediction^1^ suggested that the GroES-like domain of *Kg*BDH is located at amino acids 1–177, and the enzyme is a medium-chain alcohol dehydrogenase containing a GXXGXXG cofactor binding motif (Bottoms et al., 2002). The conserved zinc-binding site GHEXXGXXXXX[GA]XX[IVAC], which is often present in the medium-chain zinc-containing alcohol dehydrogenase family, was found in *Kg*BDH (González et al., 2001). However, the active-site motif YXXXK, which is usually present in the short-chain alcohol dehydrogenase superfamily, was absent in *Kg*BDH (Zhang et al., 2014).

### Enzymatic Characteristics of the Functional Enzyme

**FIGURE 2:**
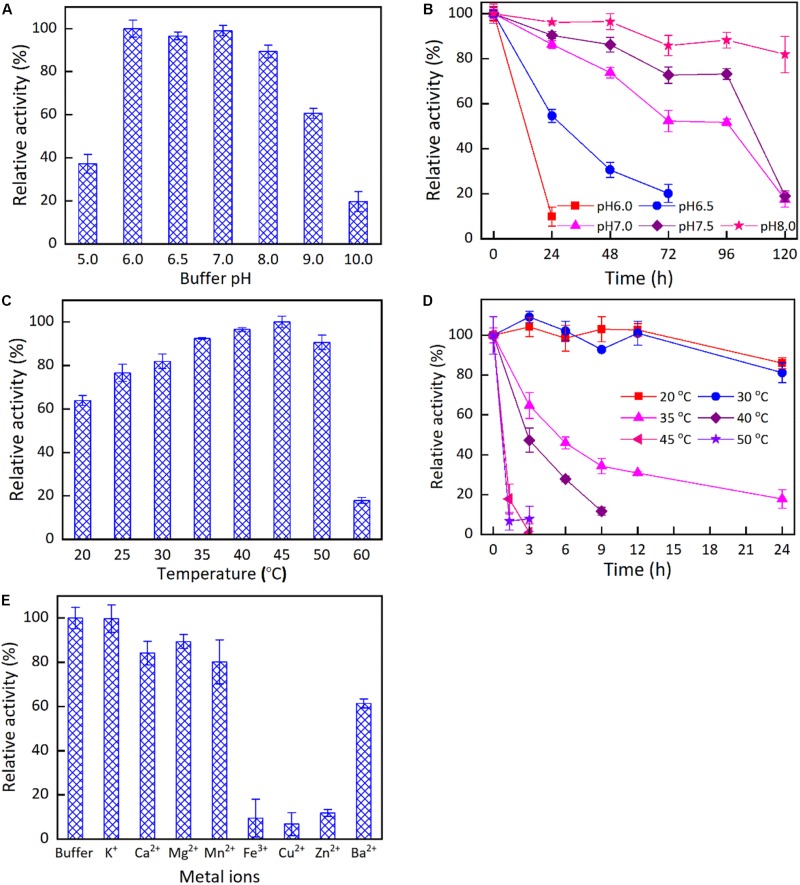
The effects of pH, temperatures and metal ions on the catalytic properties of *Kg*BDH. The reduction activity **(A)** and stability **(B)** of *Kg*BDH at different pH. The reduction activity **(C)** and stability **(D)** of *Kg*BDH at different temperatures. The reduction activity of *Kg*BDH at different metal ions **(E)**.

The impacts of buffer pH, reaction temperature, and metal ions on the activity of *Kg*BDH for the reduction of HAP were studied (Figure 2). *Kg*BDH presented good catalytic activity at pH 6.0–8.0, and the changes in the relative activity within the pH range examined were slight (Figure 2A). The pH stability assays demonstrated an enhanced trend in the residual catalytic activity of *Kg*BDH with an increase in buffer pH when the pH was varied from 6.0 to 8.0 (Figure 2B). For instance, the residual activity of *Kg*BDH remained at approximately 73 and 88% after 96-h incubation at pH 7.5 and 8.0, respectively. Figure 2C describes the effect of temperature on the catalytic activity of *Kg*BDH. The highest catalytic activity was achieved at 45°C, and an increase in temperature resulted in significantly lower activity, especially at 60°C. In terms of the thermal stability of *Kg*BDH, relatively excellent reduction activity was observed at temperatures of 20–30°C, at which more than 80% catalytic activity was retained within 24 h. However, as the incubation temperatures increased above 30°C, a rapid decline in the catalytic activity was observed during the incubation. For instance, less than 40 and 20% catalytic activity were retained after 9-h incubation at 35 and 40°C, respectively. The results suggest that the enzyme is more suitable for application at room temperature. Figure 2E shows the impact of metal ions on the catalytic activity of *Kg*BDH. The examined metal ions had no activating effect on the functional enzyme. Additionally, three metal ions, Cu^2+^, Fe^3+^, and Zn^2+^, visibly decreased the reduction activity of *Kg*BDH. Table 1 shows the kinetic parameters of *Kg*BDH on the reduction of HAP. The apparent *K*_m_ and *k*_cat_ were 5.4 mM and 4.9 s^–1^, respectively, and the *k*_cat_/*K*_m_ was 0.9 s^–1^ mM^–1^. The reduction activity of the enzyme for HAP was 6.7 U/mg. Several enzymes with high enantioselectivity for the fabrication of chiral PED by the reduction of HAP were shown in Supplementary Table S3. Compared with other functional enzymes, *Kg*BDH showed excellent performances in enantioselectivity or reduction activity.

**TABLE 1:** Kinetic parameters of *Kg*BDH for the reduction of HAP.

Enzyme	K_m_ (mM)	K_cat_ (S^–^^1^)	K_cat_/K_m_ (S^–^^1^/mM)	Activity (U/mg)
*Kg*BDH	5.4	4.9	0.9	6.7

### Characteristics of the Immobilized Cells

**FIGURE 3:**
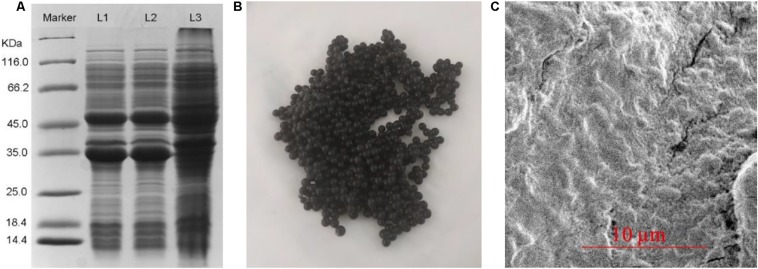
SDS-PAGE of coexpression of *Kg*BDH and *Bs*GDH in the engineered *E. coli* **(A)** and the immobilized cells **(B)** as well as their SEM **(C)**. L1: Total proteins from BL21(DE3)-pETduet1-*Kg*BDH-*Bs*GDH strain with 0.1 mM IPTG. L2: Soluble proteins from BL21(DE3)-pETduet1-*Kg*BDH-*Bs*GDH strain with 0.1 mM IPTG. L3: Total proteins from BL21(DE3)-pETduet1-*Kg*BDH-*Bs*GDH strain without IPTG.

As depicted in Figure 3A, SDS-PAGE analysis indicated that *Kg*BDH and *Bs*GDH were successfully coexpressed in *E. coli* BL21(DE3). Figures 3B and C show the appearance and micrograph of the immobilized *E. coli* BL21(DE3)-*Kg*BDH-*Bs*GDH cells by the mixture of calcium alginate and activated carbon. The SEM micrograph in Figure 3C shows the cells entrapped in the beads. First, we compared the catalytic rates of the whole cells, the immobilized cells by calcium alginate, and the immobilized cells by the mixture of calcium alginate and activated carbon. To better calculate the produced (*R*)-PED, the adsorption models of PED in the calcium alginate beads and the activated carbon–calcium alginate beads were established. As shown in Supplementary Figure S4, the constructed Freundlich adsorption models adequately represent the adsorption of PED in the beads. The reaction catalytic rate of the cells embedded in the activated carbon–calcium alginate beads, compared with that of free whole cells, showed a slight difference, whereas a visible decline in the catalytic rate of the cells embedded in the calcium alginate beads was observed (Supplementary Figure S3c). Moreover, the cells in the activated carbon–calcium alginate beads showed a 1.8-fold higher reaction rate than that in the calcium alginate beads. Prior report has indicated that *Paracoccus* sp. strain KT-5 immobilized by activated carbon degraded pyridine with a higher reaction rate than that of the free cells (Qiao et al., 2010). Additionally, regardless of the adsorption capacity of activated carbon, the degradation time of pyridine at 1476 mg/ml by the immobilized cells was shorter than that at 978 mg/ml by free cells. Mörsen and Rehm (1990) have reported that microbial cells immobilized by activated carbon displayed a more rapid phenol-degrading rate than those immobilized by sintered glass, probably because of the rapid adsorption of phenol by activated carbon. Our previous work has also indicated that the catalyst with better adsorption hydrolyzes cellobiose more rapidly to form glucose (Hu et al., 2014). We therefore speculated that the adsorption effect of active carbon might be responsible for the stimulation, because the rapid adsorption was beneficial in increasing the effective contact between the cells and the substrate.

**FIGURE 4:**
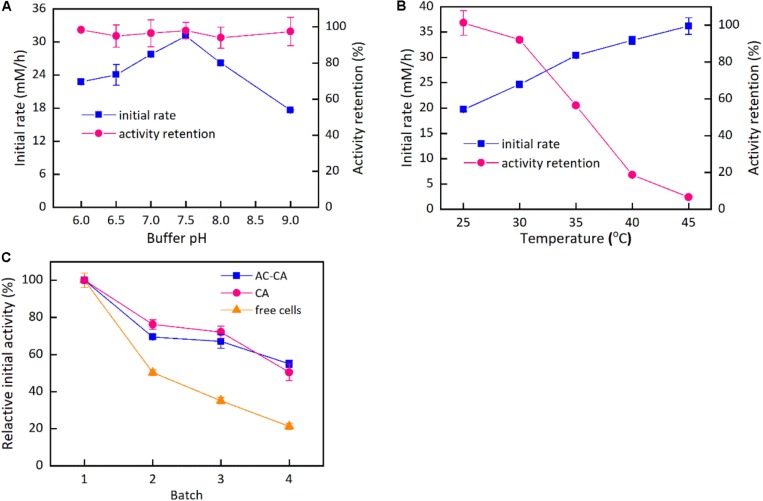
Effect of buffer pH and temperature on the immobilization cells and their operation stability. Reaction conditions: 20 mM HAP, 40 mM Glucose, 180 rpm, 25 mg/ml wet cells in beads. **(A)** 35°C, sodium acetate (100 mM, pH 6.0–7.0) and Tris–HCl (50 mM, pH 7.5–9.0); **(B)** 25–45°C, Tris–HCl (50 mM, pH 7.5–9.0); **(C)** 35°C, Tris–HCl (50 mM, pH 7.5–9.0).

Figure 4 describes the effects of buffer pH and reaction temperature on the catalytic activity of the immobilized cells as well as operational stability. As shown in Figure 4A, the impacts of buffer pH on the catalytic activity and stability of the cells immobilized by the mixture of activated carbon and calcium alginate were studied. The buffer pH exhibited an important influence on the catalytic activity of the immobilized cells. The catalytic rate was enhanced with an increase in buffer pH from pH 6.0 to the maximum at pH 7.5. Continuously increasing the buffer pH led to a sharp decline in the catalytic activity. Figure 4A also indicates that the immobilized cells performed excellent pH stability within the pH range from 6.0 to 9.0. Slight differences in the catalytic rate of the immobilized cells were observed after 12-h incubation in the buffer of various pH values, thus indicating that the immobilized cells could be stored in a wide pH range. The initial reaction rate of the immobilized cells continued to increase when the temperature was varied from 25 to 45°C (Figure 4B). However, the thermal stability assay of the immobilized cells indicated that the residual catalytic activity rapidly decreased as the temperature increased from 25 to 45°C. In comparison, the residual catalytic activities after 12-h incubation at 25 and 30°C remained at approximately 100% and above 90%, respectively; nevertheless, when the incubation temperature was 35 and 40°C, the catalytic activities remained at approximately 56 and 19% after 12 h, respectively. The results suggest that the immobilized cells are suitable for the reaction temperatures ≤35°C. As shown in Figure 4C, the two immobilized cells had better operational stability than the free cells. The two immobilized cells had similar trend in the operational stability. The cells immobilized by the mixture of calcium alginate and activated carbon retained approximately 55% of the initial catalytic activity after four reuse cycles, a level higher than that of free cells, which retained approximately 20% of their initial catalytic activity.

### Synthesis of (*R*)-PED by a Fed-Batch Strategy

**FIGURE 5:**
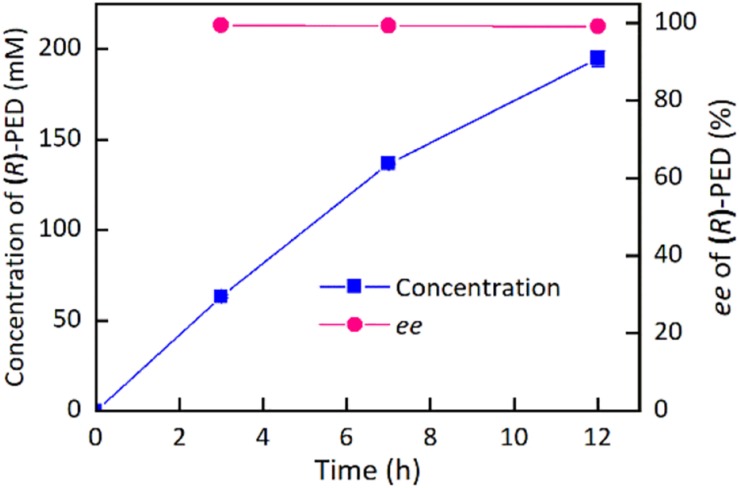
Preparation of (*R*)-PED by a fed-batch strategy.

On the basis of the above results, the buffer pH and reaction temperature for the reduction of HAP by the immobilized cells were chosen at 7.5 and 35°C, respectively. Supplementary Figure S5 demonstrates the impact of HAP concentration on the synthesis of (*R*)-PED. The HAP concentration was evaluated at 80 mM, given the high expression level of target protein in the engineered strain. As shown in Supplementary Figure S5a, 83–86% of (*R*)-PED yields were achieved at the end of the reactions when the HAP concentrations were 80–100 mM. In addition, excellent enantioselectivities (>99%) were observed. However, when the HAP concentration reached 150 mM, part of HAP was insoluble, which would decrease the reaction efficiency. We also used DMSO as a cosolvent (Cui et al., 2017) to improve the solubility of HAP; on the other hand, 150 mM HAP was still not completely dissolved in the reaction systems containing 10 or 15% DMSO (v/v) (Supplementary Figure S5b). We then employed fed-batch feeding of HAP to increase the concentration of the substrate (Figure 5). Approximately 195 mM (*R*)-PED (81% yield) was produced after two-batch feeding of HAP within 12 h, and the optical purity of (*R*)-PED was >99%. A productivity of approximately 54 g/L per day was achieved. In comparison to the fabrication of (*R*)-PED from the reduction of HAP by microbial cells, as shown in Table 2, our preparation approach provided some advantages, such as cofactor regeneration, easy reuse, and even a high substrate level. In this study, in comparison to Cui’s work, the catalytic reaction had some shortcomings, such as lower substrate loading and product yield. However, the biocatalyst loading in our study was significantly less than that in Cui’s work (25 mg/ml wet cells versus 30 mg/ml lyophilized cells), and the immobilized cells were more easily reused than the free cells, thus making our system competitive. The improvement in HAP solubility in the reaction system is key in enhancing reaction efficiency. Some studies (Liese et al., 1996; Peng et al., 2020) have found that HAP is easily soluble in organic solvents. The use of organic solvents efficiently improved the substrate concentration and eased the inhibition effect of HAP to whole cell biocatalysts. The delightful results suggested that the application of an organic-aqueous two-liquid phase system may increase the substrate concentration in the asymmetric reduction of HAP for fabrication of (*R*)-PED by *E. coli* BL21(DE3)-*Kg*BDH-*Bs*GDH cells.

**TABLE 2:** The preparation of (*R*)-PED by asymmetric reduction of HAP using biocatalytic approach.

Strains	Cells loading	Substrate (mM)	Time (h)	Yield (mM)	*ee* (%)	References
*E. coli* BL21(DE3)-*Kg*BDH-*Bs*GDH	Immobilized beads including 25 mg/ml wet cells	240	12	195	>99	This study
*E. coli* RIL/pET-R-SD-AS-G	50 mg/ml wet cells	44	24	44	99.9	Zhou et al., 2015
*Candida parapsilosis*	10 mg/ml wet cells	74	5	≈55	99.9	Wang et al., 2012
*E. coli* BL21(DE3)-BDHA-GDH	30 mg/ml lyophilized cells	400	3	396	>99	Cui et al., 2017

## Conclusion

*Kg*BDH from *K. gibsonii* SC0312 was capable of reducing HAP to (*R*)-PED in high enantioselectivity. A constructed recombinant *E. coli* BL21(DE)-*Kg*BDH-*Bs*GDH strain was used to produce (*R*)-PED through the asymmetric reduction of HAP by using glucose as a cosubstrate. *E. coli* BL21(DE)-*Kg*BDH-*Bs*GDH cells immobilized by the mixture of activate carbon and calcium alginate showed a 1.8-fold improvement in the initial rate over that of cells immobilized by calcium alginate. Furthermore, high levels of HAP (240 mM) were reduced to (*R*)-PED by *E. coli* BL21(DE)-*Kg*BDH-*Bs*GDH cells immobilized by the mixture of activate carbon and calcium alginate by a fed-batch strategy, affording 195 mM yield and >99% *ee*. Further studies should focus on the improvement of HAP concentration for the asymmetric reduction of HAP to (*R*)-PED by *E. coli* BL21(DE)-*Kg*BDH-*Bs*GDH cells.

## Data Availability Statement

The datasets generated for this study can be found in the article/Supplementary Material.

## Author Contributions

FP, W-YL, and M-HZ conceived and designed the experiments. FP and X-YO performed the experiments. FP and H-HS analyzed the data. FP, Z-FN, and W-YL wrote the manuscript.

## Conflict of Interest

The authors declare that the research was conducted in the absence of any commercial or financial relationships that could be construed as a potential conflict of interest.

## Footnotes

1 http://www.ebi.ac.uk/Tools/InterProScan/
