# Supplementary material for: Immobilization of Cofactor Self-Sufficient Recombinant Escherichia coli for Enantioselective Biosynthesis of (R)-1-Phenyl-1,2-Ethanediol
Source: Front Bioeng Biotechnol. 2020 Feb 21;8:17. doi: 10.3389/fbioe.2020.00017 (PMC7046757; doi:10.3389/fbioe.2020.00017)
Supplement: Supplementary file 1 [file Data_Sheet_1.pdf]

Supplementary Material

The complement of experimental methods

1 Clone, expression of genes and enzymatic purification

Based on the gene sequences of *K. gibsonii* 11kri321 strain in NCBI database, we choose eight dehydrogenases and designed the corresponding primers (Table S1), which were synthesized by Sangon Biotech (Shanghai, China). The eight putative dehydrogenase genes were amplified by PCR from *K. gibsonii* SC0312. The PCR products were digested by restriction enzyme and subsequently inserted into the expression vector pET28a which had been digested using the same enzymes. The ligation mixture was transformed into competent *E. coli* DH5α cells. The positive clones were confirmed by extraction of plasmid and verification of gene sequencing. The recombinant plasmids were then transformed into *E. coli* BL21 (DE3) competent cells for gene expression.

The recombinant *E. coli* BL21 (DE3) cells were cultured in Luria-Bertani (LB) broth containing 100 ug/mL Kanamycin at 37 °C and 160 rpm overnight, and then 1 mL seed solution was inoculated into 100 mL fresh LB broth containing Kanamycin (50 mg/L) to grow at the same culture conditions. When the OD600 of the cell cultures reached 0.6-0.8, the injection of IPTG (0.1 mM) induced the expression of the target genes, followed by incubation at 20 °C and 160 rpm for 20-24 h. The cells were collected by centrifugation (6000*g*, 3min) at 4 °C and washed twice by normal saline.

The above collected cells were dissolved in the phosphate buffer (PB, 50 mM, pH7.4) and smashed by sonication. Subsequently, the crude extract was centrifuged to achieve the supernatant at 10000*g* and 4 °C for 15 min. Ni-NTA agarose column was employed to purify recombinant enzymes in the supernatant. The purified enzyme was further desalted by desalting column. The genes expression and purification of enzymes were verified by SDS-PAGE.

2 Construction of the engineered strain with co-expression of *Kg*BDH and *Bs*GDH

Glucose dehydrogenase (*Bs*GDH) gene from *Bacillus subtilis* 168 strain and *Kg*BDH gene were amplified by PCR using the primers in Table S2. In the first step, *Bs*GDH gene was inserted to pETduet1 plasmid. The PCR products of *Bs*GDH gene were digested by restriction enzyme and subsequently ligated into the pETduet1 which had been digested using the same enzymes. The ligation mixture was transformed into competent *E. coli* DH5α cells. The positive clones were confirmed by extraction of plasmid and verification of gene sequencing. In the second step, *Kg*GDH gene was inserted to the recombinant pETduet1 above. The PCR products of *Kg*BDH gene were digested by BgI II and Xho l, and subsequently ligated into the recombinant pETduet1 which had been digested using the same enzymes. The ligation mixture was transformed into competent *E. coli* DH5α cells. The positive clones were confirmed by extraction of plasmid and verification of gene sequencing. The recombinant plasmids containing *Kg*BDH gene and *Bs*GDH gene (pETduet1-*Kg*BDH-*Bs*GDH) were then transformed into *E. coli* BL21 (DE3) competent cells.

Table S1. The primers for the clone of predicted genes

Entry	Gene ID	Enzyme	Primer sequence		Restriction sites
1	WP_068452693	*Kg*IDH	Primer-F	5'—CGCCATATGAAAGCAGCAGTAGTCGCAAG—3'	Nde I
			Primer-R	5'—CCGGAATTCTTTTGTAAAGTCAATTACCATACGTCCT—3'	EcoR I
2	WP_068454545	*Kg*ADH1	Primer-F	5'—CGCCATATGGAGTATAGAAACTTAGGTCC—3'	Nde I
			Primer-R	5'—CCGGAATTCTTTCATACGATGTTCTTCGTAT—3'	EcoR I
3	WP_068457222	*Kg*AKR	Primer-F	5'—CGCCATATGCCACGTTTTGGTTTAGGT—3'	Nde I
			Primer-R	5'—CCGGAATTCTTTTTTCAGATGGAATGTATCC—3'	EcoR I
4	WP_068450250	*Kg*LAH	Primer-F	5'—CTAGCTAGCATGGAAAATTTTAAGTATCAAAACC—3'	Nhe l
			Primer-R	5'—CGCGGATCCTAATGAACGTTTTAAAATTTCTTTT—3'	BamH I
5	WP_068452021	*Kg*ADH2	Primer-F	5'—CTAGCTAGCATGAAAGCGGTTTATGCAGA—3'	Nhe l

			Primer-R	5'—CGCGGATCCCATCGTTAATACTATTTTGCCAAAC—3'	BamH I
6	WP_068453520	*Kg*OR	Primer-F	5'—CTAGCTAGCATGAAGACAATTGAAGTACATGC—3'	Nhe l
			Primer-R	5'—CGCGGATCCTACGAGTTGTTCAAGTTCTTGC—3'	BamH I
7	WP_068453534	*Kg*ADH3	Primer-F	5'—CTAGCTAGCATGTTTAACGAAAAGTACAAATTATC—3'	Nhe l
			Primer-R	5'—CGCGGATCCTTTAATTTTGCCACCGAAAAC—3'	BamH I
8	WP_068456444	*Kg*BDH	Primer-F	5'—CGCGGATCCATGAAAGCAGCAGTATGGTATG—3'	BamH I
			Primer-R	5'—CCGGAATTCAAATTCTCCACTTAATTCTACAAG—3'	EcoR I

Table S2. The primers of *Kg*BDH and *Bs*GDH for the construction of whole-cell biocatalyst

Entry	Enzyme	Primer name	Primer sequence	Restriction sites
1	*Kg*BDH	Primer-F	5'-GGAAGATCTCATGAAAGCAGCAGTATGGTATGGT-3'	BgI II

		Primer-R	5’-CCGCTCGAGTTAAAATTCTCCACTTAATTCTACAAGAATT-3’	Xho l
2	*Bs*GDH	Primer-F	5'-TGCACTGCAGATGTCCCCTATACTAGGTTATTGGG-3'	Pst I
		Primer-R	5'-GCGTCGACTTATTGCGCGGTGTACCCACCATCAAT-3'	Sal I

Table S3. The comparison of function enzymes capable of asymmetrically reducing HAP.

Entry	Enzyme	*ee* (%)	Activity (U/mg)	Optimal pH	References
1	*Kg*BDH	>99% (*R*)	6.7	pH 6-8	This study
2	CMCR	99% (*S*)	3.5	---	[1]
3	CprCR	80.7% (*R*)	1.35	pH 7-8	[2]
4	BDHA	>99% (*R*)	2.1	pH 6-8	[3]

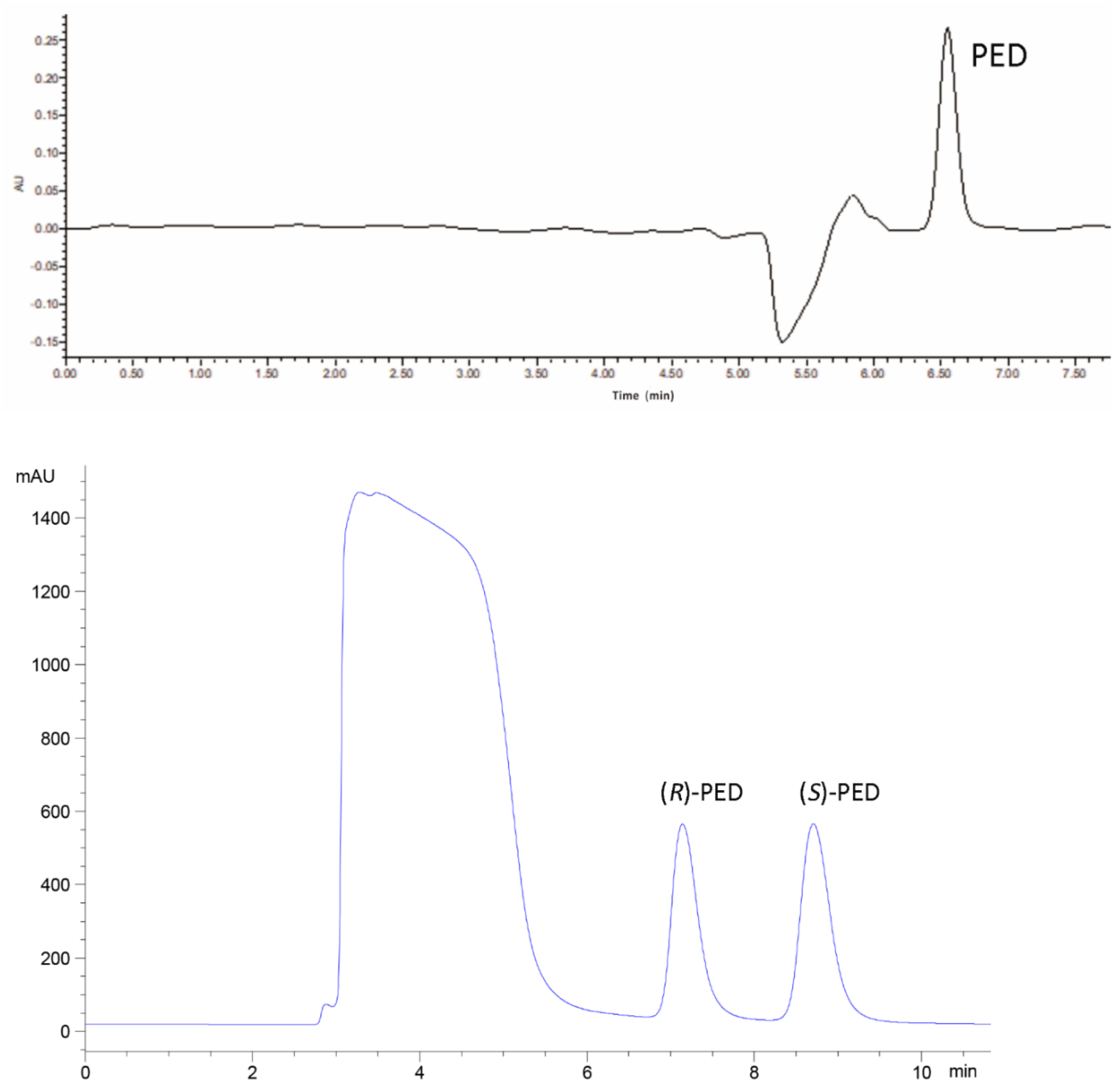

Figure S1. The Waters HPLC chromatogram of PED (a) as well as the Agilent HPLC 1100 chromatogram of (*R*)-PED and (*S*)-PED (b).

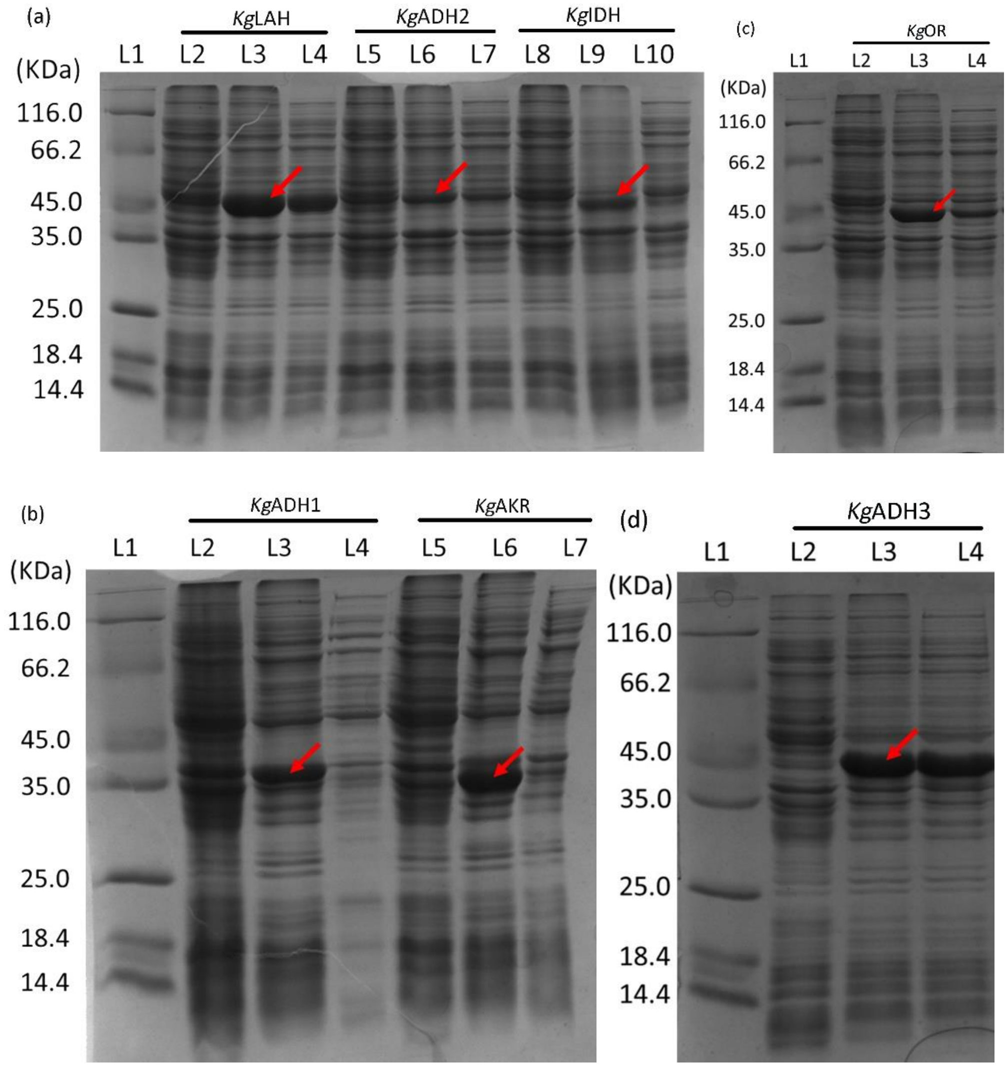

Figure S2. SDS-PAGE of proteins. Marker: L1, (a) Control: L2, 5, 8; Whole cell: L3, 6, 9; Supernatant: L4, 7, 10; (b) Control: L2; Whole Cell: L3; Supernatant: L4; (c) Control: L2, 5; Whole cell: L3, 6; Supernatant: L4, 7; (d) Control: L2; Whole Cell: L3; Supernatant: L4. Control, total proteins from a recombinant strain without IPTG; Whole cell, total proteins from a recombinant strain with 0.1 mM IPTG; Supernatant, Soluble proteins from a recombinant strain with 0.1 mM IPTG.

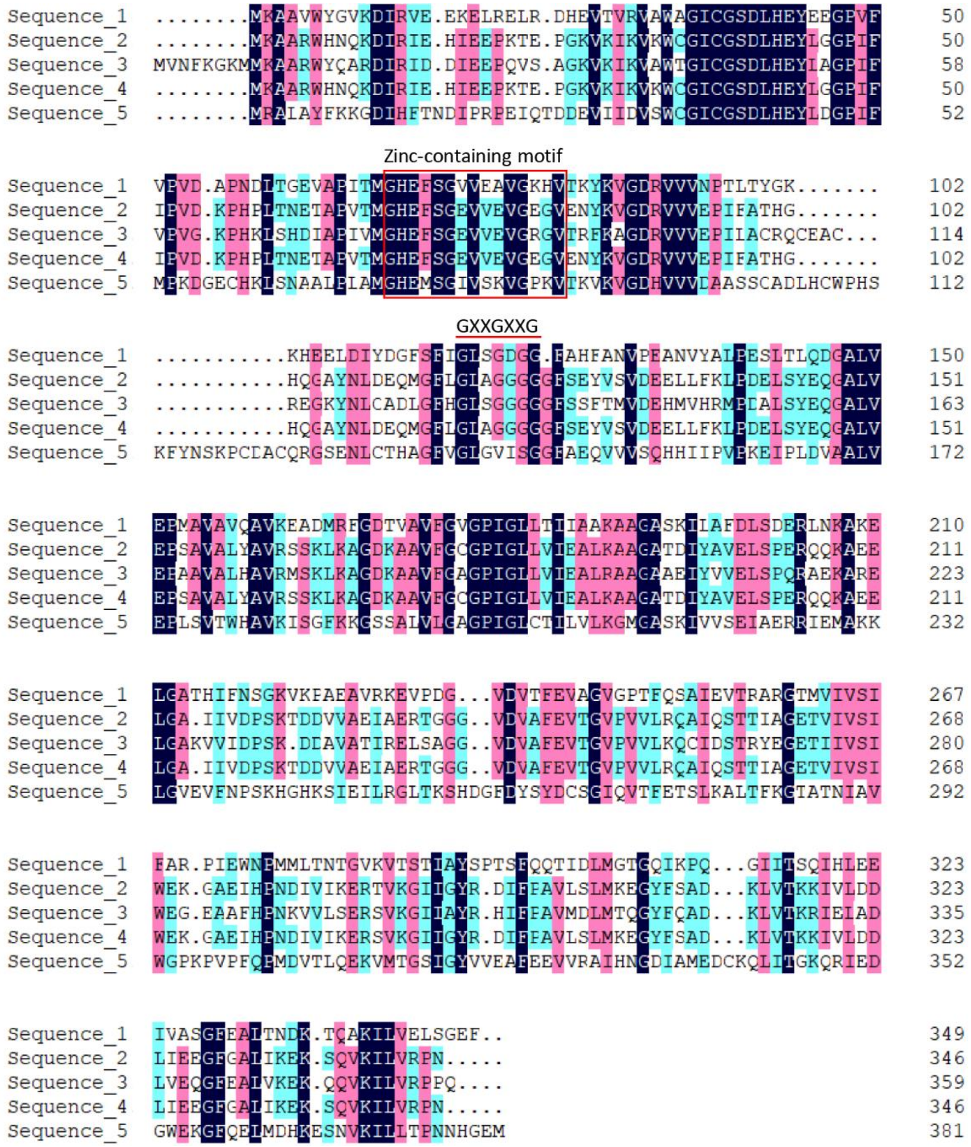

Figure S3. Amino acid sequence multiple alignment of *Kg*BDH with other (2R,3R)-2,3-butanediol dehydrogenases. Sequence 1: *Kg*BDH; Sequence 2: AOA09926.1; Sequence 3: AEF51265.1; Sequence 4: JN387994.1; Sequence 5: AAC04974.1.

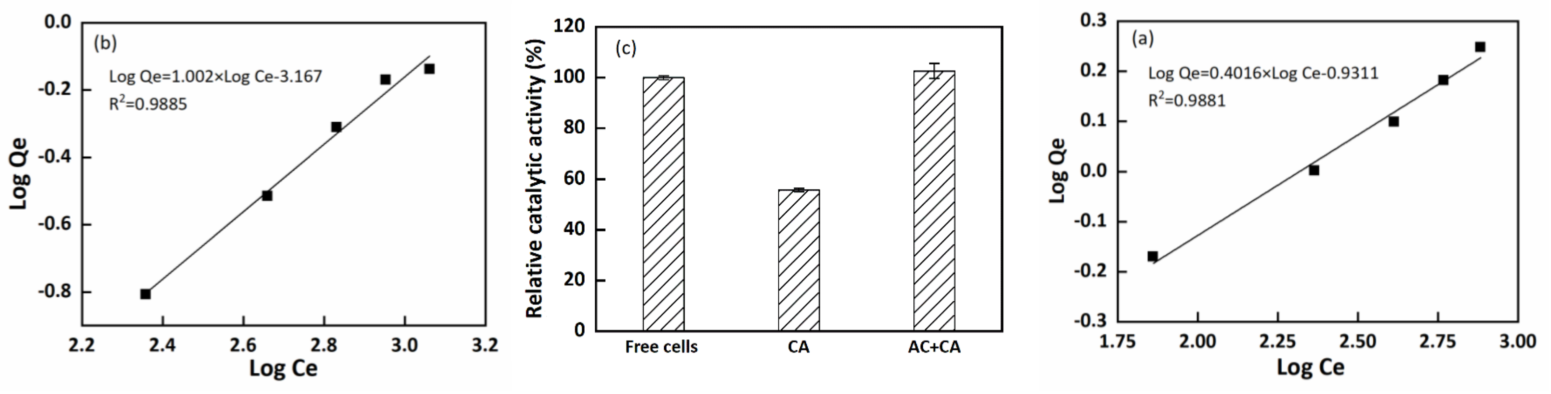

Figure S4. The Freundlich adsorption model of PED in calcium alginate beads (a) and activated carbon-calcium alginate (b); and the comparison of catalytic activity of BL21(DE3)-pETduet1-*Kg*BDH-*Bs*GDH cells (Free cells), the cells immobilized by calcium alginate beads (CA) and the cells immobilized by the mixture of activated carbon and calcium alginate (AC+CA), respectively (c). Qe, the amount of PED in beads, mg/g; Ce, the amount of PED in buffer, mg/L.

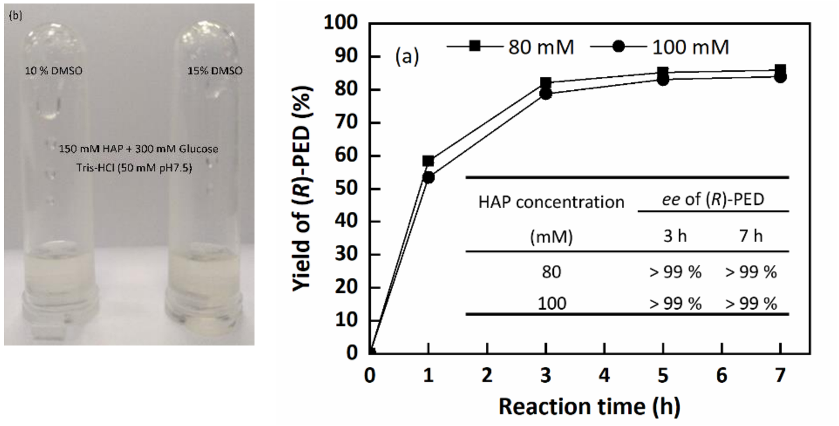

Figure S5. The effect of PED concentration on the catalytic performances of immobilized cells (a); Insoluble HAP in the buffer with 10% and 15% DMSO (v/v).

References

[1] Chen X, Mei T, Cui Y, Chen Q, Liu X, Feng J, Wu Q, Zhu D. Highly efficient synthesis of optically pure (*S*)-1-phenyl-1,2-ethanediol by a self-sufficient whole cell biocatalyst. ChemistryOpen 2015; 4: 483-488.

[2] Nie Y, Xu Y, Wang HY, Xu N, Xiao R, Sun ZH. Complementary selectivity to (*S*)-1-phenyl-1,2-ethanediol-forming *Candida parapsilosis* by expressing its carbonyl reductase in *Escherichia coli* for (*R*)-specific reduction of 2-hydroxyacetophenone. Biocatal Biotransform; 2008, 26: 210-219.

[3] Cui ZM, Zhang JD, Fan XJ, Zheng GW, Chang HH, Wei WL. Highly efficient bioreduction of 2-hydroxyacetophenone to (*S*)- and (*R*)-1-phenyl-1,2-ethanediol by two substrate tolerance carbonyl reductases with cofactor regeneration. J Biotechnol 2017; 243: 1-9.
